# Seasonal Variation and Global Public Interest in the Internet Searches for Osteoporosis

**DOI:** 10.1155/2021/6663559

**Published:** 2021-06-04

**Authors:** Chao Wang, Xiong Shu, Jianfeng Tao, Yanzhuo Zhang, Yue Yuan, Chengai Wu

**Affiliations:** ^1^Department of Molecular Orthopaedics, Beijing Research Institute of Traumatology and Orthopaedics, Beijing Jishuitan Hospital, Beijing, China; ^2^Department of Epidemiology and Biostatistics, Beijing Research Institute of Traumatology and Orthopaedics, Beijing Jishuitan Hospital, Beijing, China; ^3^Bone Tissue Bank, Beijing Research Institute of Orthopaedics and Traumatology, Beijing Jishuitan Hospital, Beijing, China

## Abstract

**Background:**

To ascertain the seasonal pattern and global public interest in osteoporosis by evaluating search term popularity changes of the disease over a decade.

**Methods:**

We applied Google Trends to retrieve search popularity scores for the term “osteoporosis” between January 01, 2004, and December 31, 2019. Cosinor analyses were conducted to examine the seasonality of osteoporosis, and analysis on osteoporosis-related topics including hot topics and rising-related topics was also performed.

**Results:**

The cosinor analyses demonstrated a statistically significant seasonal variation in relative search volume of the “osteoporosis” in the world (*p* = 0.0083), USA (*p* < 0.001), UK (*p* < 0.001), Canada (*p* < 0.001), Ireland (*p* < 0.001), Australia (*p* < 0.001), and New Zealand (*p* < 0.001), with a peak in the late winter months and trough in the summer months. The peaks in late winter and valley in summer presented an approximately 6-month difference between hemispheres. The top 11 rising topics were denosumab, FRAX, hypocalcaemia, zoledronic acid, ibandronic acid, osteomyelitis, osteopenia, osteoarthritis, bone, calcium, and bone density.

**Conclusions:**

Google search query volumes related to osteoporosis follow strong seasonal patterns with late winter peaks and summer troughs. Further studies aimed at elucidating the possible mechanisms behind seasonality in osteoporosis are needed. Moreover, Internet data including the top rising topics may alert physicians to strengthen the propaganda of osteoporosis timely, so as to further promote the development of public health interventions.

## 1. Introduction

Osteoporosis is defined as a skeletal disease characterized by low bone mass, deterioration of bone tissue, and disruption of bone microarchitecture, with a consequent increase in bone fragility and susceptibility to fracture [1]. Due to its prevalence worldwide, osteoporosis is considered a major public health problem. It was reported that approximately one-tenth of individuals over the age of 50 and up to one-fourth of individuals aged more than 80 years are osteoporotic in the United States [2, 3]. Because of the systemic nature of osteoporosis, the associated increase in fracture risk affects virtually all skeletal sites [4]. The World Health Organization (WHO) report emphasized that musculoskeletal conditions including osteoporotic fractures exert a heavy burden on patients and lead to important health consequences. Previous studies showed that the number of patients with osteoporotic hip fractures was about more than 200 million in the world [5, 6]. In the UK, one in two women and one in five men aged more than 50 years will experience an osteoporotic fracture in their lifetime. Furthermore, each year, an estimated 1.5 million individuals suffer osteoporotic fractures in the USA. Osteoporosis-related fractures impose a substantial burden of costs, which is estimated to be £4 and $17.9 billion per annum in the UK and USA, respectively [7]. The burden will increase sharply with the increasingly aging society [8].

In view of the global health impact of osteoporosis, evaluating the global public interest in this disease is in urgent need. The Internet has emerged as a reliable tool to retrieve health-related information. Internet users can search for information about disorders, medication, and treatments. Actually, Eysenbach had coined the term “infodemiology” in 2002. The “infodemiology” was described as containing the study of the determinants and distribution of health information and misinformation [9, 10]. Eysenbach indicated that finding and understanding what people are searching for online could be useful in guiding health professionals and patients to quality health information and providing information for the field of public health. He demonstrated efficient predictive power of Internet searches to predict flu outbreaks in subsequent research [11, 12]. Google Flu Trends was inspired by this excellent work [13, 14]. Nowadays, the analysis of Google search queries may represent a powerful tool to investigate human behavior on health-related topics and detect the real-time global activity of disease, including osteoarthritis (OA), rheumatoid arthritis (RA), systemic lupus erythematosus (SLE), psoriasis, and cardiovascular disease (CVD) [15–19]. The use of Google Trends in analyzing health-related information might found new clues for health policy makers by estimating health-related needs and behaviors. However, an effective way to measure the global public interest in osteoporosis has yet to be determined.

To extend the work of these previous studies, we performed Google Trends to track seasonal patterns and investigate global public interest in osteoporosis.

## 2. Methods

### 2.1. Google Trends Interrogation and Data Gathering

The Google Trends provides data for Internet search activity regarding the frequency of search terms. To perform easier comparisons between different terms, Google Trends uses a scale from 0 to 100 to represent the relative search volume (RSV) of a search term for the time and geographic areas. To eliminate selection bias, Google Trends excludes duplicate queries by the same person in a short time [20]. We searched the term “osteoporosis” worldwide with the “health” category during the period of January 01, 2004, and December 31, 2019. And the search was also conducted in majority native English-speaking countries including four northern hemisphere countries (USA, UK, Canada, and Ireland) and two southern hemisphere countries (Australia and New Zealand). The monthly data were downloaded from Google Trends in Comma-Separated Values (CSV) format to Microsoft Excel on March 5, 2020 (12 points/year × 16 years = 192 data points for each country). The present study was performed according to the Helsinki Declaration and the policy of Google. The database was sourced totally from the Internet, and none of the queries in the database for this study can be associated with any individual [16, 20].

### 2.2. Statistical Analysis

The cosinor analysis was used to explore the seasonal variation, based on the sinusoidal patterns with the formula of

(1) $$\begin{array}{c} St=Acos\left(\frac{2\pi t}{c}-P\right),t=1,\cdots ,n, \end{array}$$

where *A* represents the amplitude of the sinusoid, which explains the size of the seasonal changes; *P* represents the phase, which explains where the seasonal peak occurs; *c* represents the length of the seasonal cycle (established at 12 for monthly data); *t* represents the time; and *n* represents the total number of data points. The cosinor analysis has a sine *p* value and cosine *p* value, and the significance level is set at *p* < 0.025 for controlling the type I error in multiple testing. One of the two values was presented (i.e., cosine *p* value). We used the Poisson model in the cosinor analysis and conducted a time series plot to display the consistency in the seasonal patterns. The details of cosinor analysis and the software utilized to execute it are described in the studies by Barnett and Dobson [21, 22]. Statistical analyses were performed by using the “season” package in R version 3.6.2.

## 3. Results

### 3.1. Osteoporosis-Related RSV from January 01, 2004, to December 31, 2019, and Seasonal Pattern

**Figure 1:**
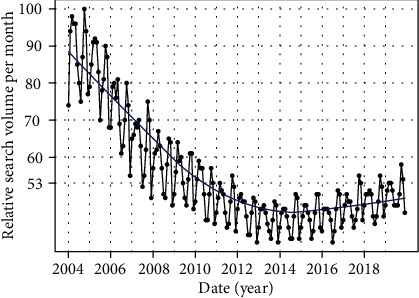
Time series plots for the relative search volume of “osteoporosis” in the world.

**Figure 2:**
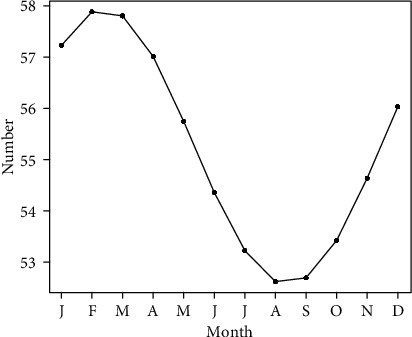
The plots of cosinor models for the seasonal variation in the relative search volume of “osteoporosis” in the world.

**Figure 3:**
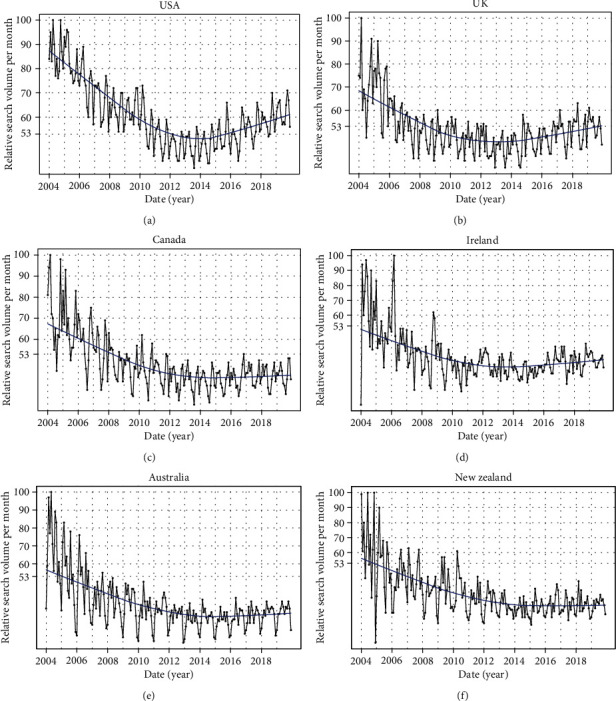
Time series plots for osteoporosis-related RSV in USA (a), UK (b), Canada (c), Ireland (d), Australia (e), and New Zealand (f) from January 01, 2004, to December 31, 2019.

**Figure 4:**
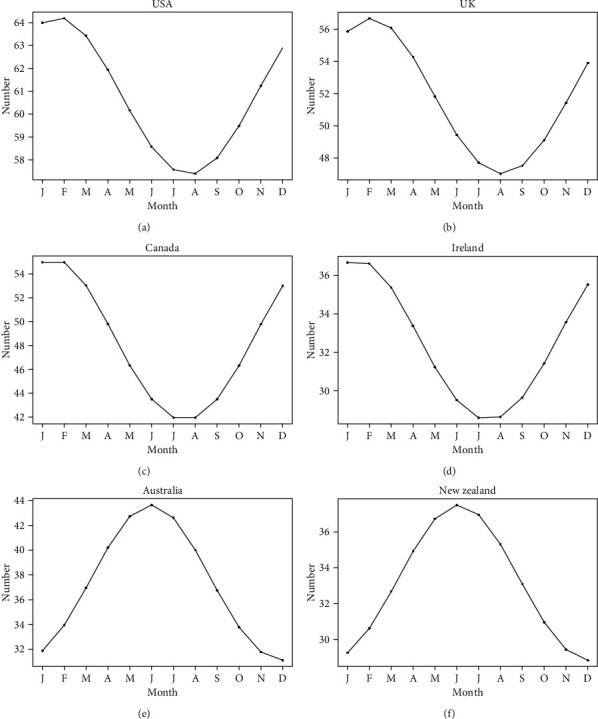
Plots of cosinor models for the seasonal patterns in osteoporosis-related RSV in USA (a), UK (b), Canada (c), Ireland (d), Australia (e), and New Zealand (f). January–December corresponding to 1–12.

Overall, RSV for osteoporosis showed a decreasing trend from January 2004 to December 2014 and then demonstrated a slowly increasing trend from January 2015 to December 2019 in the world (Figure 1). The cosinor test revealed significant seasonal variation in osteoporosis-related RSV (amplitude = 2.75; phase: month = 2.4; low point: month = 8.4; *p* = 0.0083). RSV peaked in February and reached the trough in August (Table 1 and Figure 2). Moreover, the cosinor analysis was performed for exploring the seasonal variation in the USA, UK, Canada, Ireland, Australia, and New Zealand, respectively. Of note, the peaks of osteoporosis-related RSV also occurred in late winter months (January/February for the four northern hemisphere countries and June for the two southern hemisphere countries) and reached the lowest level in the summer months (July/August for the four northern hemisphere countries and December for the two southern hemisphere countries). Time series plots for osteoporosis-related RSV and plots of cosinor models for the seasonal patterns in the six countries are presented in Figures 3 and 4, respectively.

### 3.2. Top Related Topics regarding Osteoporosis

According to GT analysis, the top related topics regarding osteoporosis were bone, osteoarthritis, calcium, osteopenia, bone density, alendronic acid, denosumab, zoledronic acid, ibandronic acid, osteomyelitis, FRAX, and hypocalcaemia (Table 2).

### 3.3. Relatively Fast-Growing Topics regarding Osteoporosis

The search term's progression was compared with the previous period. Through analyzing the relative progression of the topic osteoporosis, we found that the top rising topics were denosumab, FRAX, hypocalcaemia, zoledronic acid, ibandronic acid, osteomyelitis, osteopenia, osteoarthritis, bone, calcium, and bone density, ranking from high to low by relative growth of topics about osteoporosis (Table 3).

## 4. Discussion

The present study used Google search data to explore information-seeking behavior for osteoporosis and indicated that the number of searches for osteoporosis declined between January 2004 and December 2014 but has steadily increased since 2015. The Internet is a practical and cost-efficient health information source, and the main advantages and attractions include access, anonymity, social support, and potential for interactivity [23]. Recently, the Internet has played an increasingly important role in accessing health information and is being used more frequently in the public health promotion [14]. In 2015, a report from Google implied that one in 20 searches is to obtain health information [24]. Several studies found that more than half of US adults use Internet to seek health-related information include diagnosing or learning about a health concern [24]. Furthermore, we found that public interest in osteoporosis through Google search activity presents a seasonal pattern, with peaks in January or February for the northern hemisphere countries and June for the southern hemisphere countries. It exhibits a seasonal variation, with a peak in late winter and a nadir in summer.

Osteoporosis is prevalent and increasing musculoskeletal disorders that cause a significant burden on individuals and societies as well since they exact a societal toll in hospitalization, medical costs, loss of productivity, pain, and suffering [25]. However, the pathogenesis of osteoporosis is not yet completely clear [26]. It is interesting that several factors have distinct seasonal patterns which could partially explain development and progression of osteoporosis.

Vitamin D deficiency is a worldwide epidemic with multiple implications on human health [27]. It is worth noting that vitamin D metabolism changes with age and a decreased formation of active metabolites might be one of the causes of osteoporosis [28, 29]. Vitamin D status can be assessed by measuring the serum concentration of 25(OH)D, which shows a seasonal variation [30, 31]. Kasahara and colleagues measured vitamin D levels in 3.44 million blood samples collected in the United States and analyzed time series data spanning 287 consecutive weeks. They found vitamin D levels peaking in August and troughing in February, which might relate to intensity and exposure time of ultraviolet radiation [32]. On the other hand, patients with osteoporosis have been shown to be more likely to have VD deficiency than healthy people, and the serum 25(OH)D level also is highest at summer and at its nadir at winter [5, 33]. Furthermore, Moon and her colleagues reported that there was a significant seasonal pattern to Internet searches for vitamin D with a peak in February and nadir in August worldwide by employing the Google Trends datasets. The result was consistent with the analysis of osteoporosis in our study [34].

Cortisol is a glucocorticoid hormone produced by the adrenal glands, which have the circadian and seasonal rhythms. Cortisol concentrations did vary significantly over seasons with the lowest cortisol noted in summer and the highest cortisol found in winter [35, 36]. Nonetheless, endogenous cortisol secretion is associated with both BMD and rate of bone loss [37]. Elevated levels of cortisol directly inhibit osteoblast proliferation, differentiation, and apoptosis in various species, which could substantially blunt the bone formation process leading to lower bone density [38]. Of note, in addition to direct effects on bone cells, glucocorticoids might also suppress the production of growth hormones and gonadal steroid, further reducing bone mass [38, 39].

Moreover, physical activity is related to the development and course of osteoporosis [26]. There is increasing evidence that exercise may modestly increase bone density [40, 41]. The National Osteoporosis Foundation (NOF) strongly recommends lifelong physical activity at all ages, for both osteoporosis prevention and overall health [42]. Several studies have investigated the relationship between seasonality and physical activity, which indicated that leisure-time physical activity (LTPA) was greater in the summer and the winter was associated with significantly lower LTPA compared with the other seasons. The cyclical nature of activity level seemed to follow the same general pattern for average daily temperature and hours of daily sunshine [43, 44]. In general, physical activity levels appear to be highest in July/August and energy expenditure decreased in winter [45–47]. Haggarty et al. implied that the reduction of physical activity in winter was due to the shorter days and adverse weather conditions [48]. Additionally, it appeared to be more common that individuals conduct Internet searches when they reduce their outdoor exercise in the winter months.

Among the top fast-growing topics, “denosumab”, “FRAX”, “hypocalcemia”, “zoledronic acid”, and “ibandronic acid” were the major concerns. Denosumab is a human monoclonal antibody against the receptor activator of nuclear factor kappa-B ligand (RANKL). RANKL is a receptor expressed on the surface of cells of the osteoclast lineage, produced by osteoblasts that interact with RANK [49]. Denosumab could mimic the action of osteoprotegerin (a natural decoy receptor of RANKL), thereby blockading the RANKL-RANK interaction and inhibiting osteoclast action and bone resorption [49]. Treatment with denosumab has been found to increase BMD, and long-term therapy has been associated with a lower rate of bone fractures [50].

The Fracture Risk Assessment Tool (FRAX) was a fracture risk assessment tool for estimating individualized 10-year probability of hip and major osteoporotic fracture, which was released by the WHO [51, 52]. FRAX was launched in 2008 with eight country-specific models. Nowadays, 71 models are currently available for 66 countries covering more than 80% of the world population on the website (http://www.shef.ac.uk/frax/). It is available in 35 languages, and about 6 million calculations are performed yearly [51].

Hypocalcemia is most commonly due to vitamin D inadequacy or hypoparathyroidism or a resistance to these hormones. In addition, hypocalcemia is a known adverse effect in denosumab or bisphosphonate treatment for osteoporosis [53]. Bisphosphonates are the drugs for the treatment of osteoporosis by inhibiting osteoclastic activity and could reduce the risk of fracture. There are two subclasses of bisphosphonates: nitrogen-containing bisphosphonates (NBPs) and non-nitrogen-containing bisphosphonates (NNBPs). Zoledronic acid and ibandronic acid belong to the class of NBPs, which are the most common subclasses of bisphosphonates. NBPs inhibit the mevalonate pathway, a fundamental metabolic pathway involved in osteoclast formation and function [54, 55]. Other top rising topics included “osteomyelitis”, “osteopenia”, “osteoarthritis”, “bone”, “calcium”, and “bone density”, and these terms include symptoms, diagnosis, and accompanying diseases of osteoporosis.

The strengths of the study include using the large amount of data based on the global data and long time series of data, to explore seasonal patterns and investigate global public interest. However, several limitations should be acknowledged. First, the only Internet search engine deployed in the current investigation was Google. The available data were clearly limited to those who have access to the Internet and can use Google instead of other search engines. Nevertheless, Google Search is the mostly used search engine (over 65% of all Internet searches) [56]. Secondly, there are a variety of reasons for people to type in a term in Google. Searches might be conducted by some people other than patients with osteoporosis, who are nonetheless interested in this topic. Thirdly, the Google Trends does not supply the demographic information (e.g., by age, sex, race, educational level, place of residence, and household incomes) of the users who searched for osteoporosis. Thus, we cannot further evaluate the seasonality by stratifying specific subpopulations. For example, older adults use fewer digital applications and spend less time online than younger individuals. Interestingly, researchers also found that the sheer breadth of activities for the Internet usage among older adults is lower than that in younger adults, but for those categories that are important to them, older adults generally use the Internet quite frequently [57].

## 5. Conclusion

In summary, Google searches for osteoporosis have declined from 2004 to 2014, while they have steadily increased in recent years. Our findings also indicated that the peak of osteoporosis may occur in late winter and the lowest level is in summer. More comprehensive studies aimed at elucidating the possible mechanisms behind seasonality in the diseases are needed. Additionally, the growing interest in osteoporosis implies massive concern regarding diagnosis, accompanying diseases, and medicine employed in the treatment. Thus, Internet data may serve as a real-time surveillance tool and an alert for healthcare systems. It suggested us to strengthen the propaganda of osteoporosis and take various measures to control bone loss or increase bone strength before winter arrives, so as to further promote the development of public health interventions and the care of patients with osteoporosis.

## Figures and Tables

**Table 1:** The seasonal variation in the relative search volume of the osteoporosis.

Country	Amplitude	Phase month^∗^	Low point month^∗^	*p* value^∗∗^
Worldwide	2.75	2.4	8.4	0.0083
USA	3.54	1.7	7.7	<0.001
UK	5.07	2.1	8.1	<0.001
Canada	7.21	1.5	7.5	<0.001
Ireland	4.43	1.5	7.5	<0.001
Australian	6.8	6.0	12.0	<0.001
New Zealand	4.63	6.1	12.1	<0.001

The cosinor test was used to examine the seasonality. ^∗^Assignment of each month: January–December corresponding to 1–12. ^∗∗^The threshold of significance is adjusted at *p* < 0.025 to control the false discovery rate due to multiple testing in the cosinor analysis. The cosine *p* value is presented.

**Table 2:** Top related topics regarding the term osteoporosis.

Rank	Search topic	Relative search volume
1	Bone	100
2	Osteoarthritis	25
3	Calcium	23
4	Osteopenia	15
5	Bone density	9
6	Alendronic acid	7
7	Denosumab	5
8	Zoledronic acid	4
9	Ibandronic acid	3
10	Osteomyelitis	3
11	FRAX	2
12	Hypocalcaemia	1

**Table 3:** Relatively fast-growing topics regarding the term osteoporosis.

Rank	Search topic	% growth
1	Denosumab	Breakout
2	FRAX	Breakout
3	Hypocalcaemia	Breakout
4	Zoledronic acid	900%
5	Ibandronic acid	700%
6	Osteomyelitis	600%
7	Osteopenia	350%
8	Osteoarthritis	300%
9	Bone	200%
10	Calcium	140%
11	Bone density	70%

Note: a term's growth was compared with the previous time period. “Breakout” is used for a term search that grew by more than 5000% compared with the previous period.

## Data Availability

The database was sourced totally from Google Trends.
